# Neuroinvasive Potential of Monkeypox Virus: A 25-Year Systematic Review

**DOI:** 10.7759/cureus.77924

**Published:** 2025-01-24

**Authors:** Arham Amir, Haseeb Mehmood Qadri, Junaid Saffi, Ansab Mahmood, Nawal Nawaz, Muhammad Ahsan Asif, Eiman Amir, Alina Abed, Syed Haider Hassan, Abdul Rafay Mahmood

**Affiliations:** 1 General Surgery and Surgical Oncology, Shaikh Zayed Medical Complex, Lahore, PAK; 2 Surgery, Lahore General Hospital, Lahore, PAK; 3 Neurological Surgery, Punjab Institute of Neurosciences, Lahore, PAK; 4 General Surgery, Lahore General Hospital, Lahore, PAK; 5 General Medicine/Emergency Medicine, University Hospitals Sussex NHS Foundation Trust, Worthing, GBR; 6 Dermatology, Imperial College Healthcare NHS Trust, London, GBR; 7 Internal Medicine, Nawaz Sharif Medical College, Gujrat, PAK; 8 Internal Medicine, Jinnah Hospital, Lahore, PAK; 9 Internal Medicine, Akhtar Saeed Medical and Dental College, Lahore, PAK

**Keywords:** brain edema, brain stem, encephalitis, fatigue, headache, monkeypox, tecovirimat, thalamus, zoonoses

## Abstract

Monkeypox (MPX) is a zoonotic disease, caused by the MPX virus. Clinical symptoms and signs of the disease are similar to, but less severe than, smallpox, presenting with fever, headache, lymphadenopathy, back pain, myalgia, and skin rash. MPX can affect the central nervous system causing different complications, including encephalitis, cerebral edema, and intracranial hemorrhage. This study aimed to assess the neurological sequelae of MPX infection focusing on the available literature. An extensive data search was conducted in September 2024, covering the period from January 2000 to August 2024, using PubMed Central and Google Scholar. Preferred Reporting Items for Systematic Reviews and Meta-Analysis (PRISMA) strategy was employed along with a combination of keywords to enlist all articles with data on MPX and its neurological manifestations, diagnosis, and treatment. All open-access case reports, case series, and original research articles in the English language, providing data on confirmed cases of MPX virus infection with documented neurological manifestations involving the brain, were reviewed.

A total of 25 studies containing data on 758 patients were included in this systematic review. The mean age and standard deviation of included patients was 26.77±12.17 years, with a female predominance (65.27%). Most of the cases (87.05%) were transmitted by animal/human contact. Fatigue/malaise (34.37%) was the most common presentation, followed by fever and headache accounting for 31.27% and 29.84%, respectively. Limb weakness/numbness (20.83%) was the most common examination finding. About 26.32% had normal glucose levels and 21.05% reported raised white blood cells on CSF routine examination. T2 hyperintensities and fluid-attenuated inversion recovery (FLAIR) hyperintensities were reported on magnetic resonance imaging in 18.75% of the cases, each. Brainstem involvement, cortical, and thalamic involvement were seen in 18.60%, 16.28%, and 13.95% of cases, respectively. About 34.61% of the patients were inadvertently given antibiotics. The emerging MPX neurological involvement is alarming and requires a prompt response. The neurotropism of MPXV is still in debate and needs to be explored. Early identification and interventions by healthcare providers can significantly impact the trajectory of MPX spread.

## Introduction and background

In 2022, a global outbreak involving about 72 non-endemic nations resulted in an uprise of suspected and laboratory-confirmed monkeypox (MPX) cases, resulting in three mortalities as of July 20, 2022 [1]. Monkeypox is a zoonotic disease which is caused by the monkeypox virus (MPXV), an enveloped double-stranded DNA virus [1]. Wild animals, including primates (Mangabey monkeys), squirrels, and Gambian pouched rats are the main reservoirs of MPXV [2]. MPX can be transmitted through direct or indirect contact with infected lesions, body fluids, or respiratory droplets of infected persons and animals [3]. Common signs and symptoms of MPX are fever, skin rashes, swollen lymph nodes, chills, back pain, and exhaustion [2]. The incubation period ranges from seven to 14 days, with lesions emerging first from the oropharynx and then spreading to the whole body [3]. MPXV is categorized into the following two variants known as clades: clade I (Central African or Congo Basin clade) and clade II (Western African clade). Clade I is more virulent than clade II, with a mortality rate of one in 10 for those infected by clade I, compared to one in 100 for those infected by clade II [2,4]. MPX is endemic in sub-Saharan Africa resulting in major morbidity and mortality in individuals over 50 years of age. On July 23, 2022, the World Health Organization (WHO) declared MPXV as the highest level of alert, designating it as a public health emergency of international concern [4].

MPX can affect the central nervous system (CNS) causing different complications, i.e., encephalitis, cerebral edema, vascular congestion, and intracranial hemorrhage [5]. The neurological manifestations of MPX are not well understood with significant gaps in current research. Existing knowledge is primarily based on limited case reports, leaving the mechanisms of central nervous system involvement largely unclear. Additionally, there is insufficient data on long-term neurological outcomes, the impact of comorbidities, and effective treatments. This highlights the necessity for more thorough studies to enhance our understanding and management of these complications. This systematic review seeks to address this gap by conducting a thorough review to describe both the common and uncommon neurological clinical manifestations as well as the manifestations upon investigations, associated with MPX.

## Review

Methodology

An extensive data search was conducted in September 2024 with the time duration between January 2000 and August 2024 using PubMed Central and Google Scholar. The Preferred Reporting Items for Systematic Reviews and Meta-Analysis (PRISMA) strategy was employed to retrieve all articles on clinical and investigational manifestations of MPX infection.

Search Strategy

The search was conducted using the Boolean operators (“AND” and “OR”), with a combination of keywords to list all articles with data on monkeypox and its neurological manifestations, diagnosis, and treatment. The following combination of keywords was used: ‘Monkeypox infection’, ‘Monkeypox neurology’, Monkeypox AND Neurological’, ‘Monkeypox meningitis’, ‘Monkeypox meningoencephalitis’, Monkeypox encephalitis’, ‘Monkeypox fever’, ‘Monkeypox AND neurological manifestations’, ‘Monkeypox viral encephalitis’, ‘Monkeypox AND brain’, ‘Monkeypox AND CNS’, ‘Monkeypox AND central nervous system’, ‘Monkeypox neurotropism’, ‘Monkeypox brain involvement’, ‘Monkeypox brain infection’, ‘Monkeypox neurological symptoms’, ‘Monkeypox neurological complications’, ‘Monkeypox neuroinflammatory response’.

Inclusion and Exclusion Criteria

All case reports, case series, and research articles provided data on confirmed cases of MPXV infection in human beings with documented neurological clinical manifestations and investigations involving the brain. Only articles published in English or non-English articles with translated versions available were reviewed. Only those articles that were open-access, provided adequate information, and were published within the defined time frame were retrieved. Animal and cadaveric studies, editorials, letters to editors, conference abstracts, short communication, and non-English language studies with inadequately accessible translated versions were excluded.

Data Collection and Analysis

Data were collected by five independent reviewers who systematically screened and extracted relevant information from eligible studies. Data extraction was done using Microsoft Word and Microsoft Excel (Microsoft 365) (Redmond, WA: Microsoft Corp.). Both qualitative and quantitative variables were included; patient demographics (age, gender), diagnostic criteria, imaging findings, mode of transmission, comorbid conditions, treatment modalities (medical or surgical), presence of autoimmune conditions, and any identified genetic factors. All extracted data were imported into SPSS software version 24 (Armonk, NY: IBM Corp.) for statistical analysis. Descriptive statistics were used to summarize the data. Tables were generated to illustrate the findings.

Data Stratification

**Figure 1:**
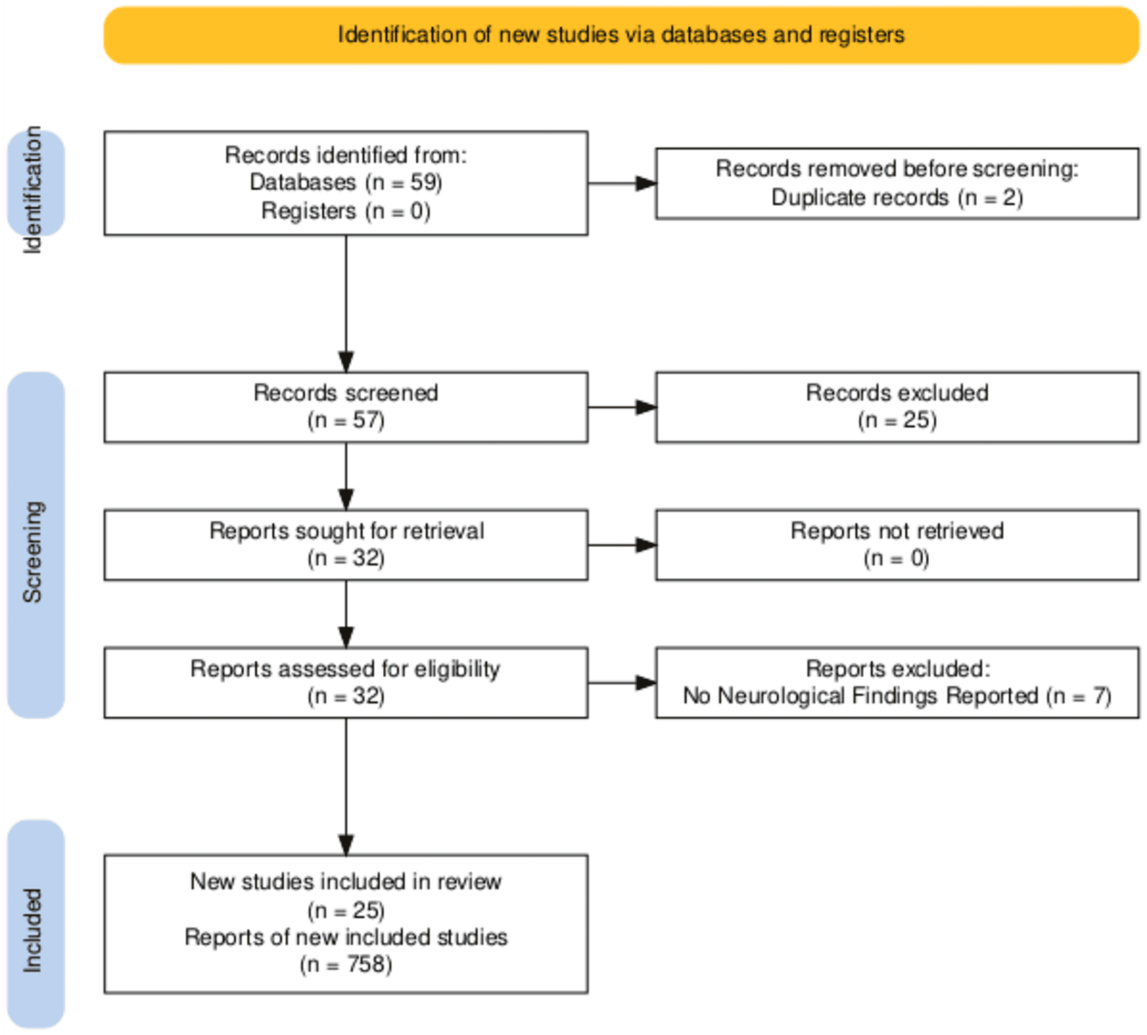
Preferred Reporting Items for Systematic Reviews and Meta-Analyses (PRISMA) flowchart for search strategy and the quality assessment of included studies.

A total of 59 articles were filtered using the keywords. Two articles were removed due to duplication and 25 studies were removed due to ineligibility. The quality of the included studies was evaluated using the Joanna Briggs Institute (JBI) critical appraisal checklist. The assessment criteria included study design, sample representativeness, case definition, the measurement of outcomes, and statistical analysis. Studies were rated according to predetermined criteria, with quality scores assigned accordingly. Further, seven articles were excluded since they did not adhere to or provide adequate findings. To summarize, a total of 25 case reports, case series, and original articles were included containing data on 758 patients in this review. A schematic PRISMA flowchart is given to illustrate the included studies (Figure 1).

Results

The details of the included literature are provided in Table 1 [6-30]. Most of the publications belonged to the United States of America, accounting for 40.0% of the total publications, followed by Congo, i.e., 16.00% (Table 2).

**Table 1:** Details of included studies.

Studies	Study title	Year of publication	Country of publication
Karin et al. [6]	Monkeypox virus-associated meningoencephalitis diagnosed by detection of intrathecal antibody production	2024	Sweden
Hammad et al. [7]	Unusual neurological complications in a patient with monkeypox: a case report	2024	Saudi Arabia
Cole et al. [8]	Monkeypox encephalitis with transverse myelitis in a female patient	2023	UK
Sharma et al. [9]	A rare co-occurrence of monkeypox encephalitis and neurosyphilis	2023	USA
Hamid et al. [10]	Manifestation of Guillain-Barre syndrome in a case of monkeypox virus infection	2023	UAE
Money et al. [11]	Monkeypox-associated central nervous system disease: a case series and review	2023	USA
Pittman et al. [12]	Clinical characterization and placental pathology of MPX infection in hospitalized patients in the Democratic Republic of the Congo	2023	Congo
Marín-Medina et al. [13]	Encephalomyelitis in a patient with monkeypox: an unusual complication	2022	USA
Pastula et al. [14]	Two cases of monkeypox-associated encephalomyelitis	2022	USA
Adler et al. [15]	Clinical features and management of human monkeypox: a retrospective observational study in the UK	2022	UK
Charniga et al. [16]	Estimating the incubation period of monkeypox virus during the 2022 multi-national outbreak	2022	USA
Akar et al. [17]	Descriptive epidemiology of monkeypox in Nigeria, September 2017-June 2019	2021	Nigeria
Hughes et al. [18]	A tale of two viruses: coinfections of monkeypox and varicella zoster virus in the Democratic Republic of Congo	2021	Congo
Ogoina et al. [19]	The 2017 human monkeypox outbreak in Nigeria - report	2019	Nigeria
Yinka-Ogunleye et al. [20]	Outbreak of human monkeypox in Nigeria in 2017-18: a clinical and epidemiological report	2019	Nigeria
Ng et al. [21]	A case of imported monkeypox in Singapore	2019	Singapore
Reynolds et al. [22]	Short report: detection of human monkeypox in the republic of the Congo following intensive community education	2013	Congo
Formenty et al. [23]	Human monkeypox outbreak caused by novel virus belonging to Congo Basin Clade, Sudan, 2005	2010	Sudan
Croft et al. [24]	Occupational risks during a monkeypox outbreak, Wisconsin, 2003	2007	USA
Reynolds et al. [25]	Clinical manifestations of human monkeypox influenced by route of infection	2006	USA
Huhn et al. [26]	Clinical characteristics of human monkeypox, and risk factors for severe disease	2005	USA
Learned et al. [27]	Extended interhuman transmission of monkeypox in a hospital community in the Republic of the Congo, 2003	2005	Congo
Reed et al. [28]	The detection of monkeypox in humans in the Western Hemisphere	2004	UK
Sejvar et al. [29]	Human monkeypox infection: a family cluster in the Midwestern United States	2004	USA
Anderson et al. [30]	A case of severe monkeypox virus disease in an American child: emerging infections and changing professional values	2003	USA

**Table 2:** Publications by countries. The "n" refers to the frequency or number of presentations. The "n/N" signifies the formula for calculating the percentage, where "N" is the total number.

Country of publication	Frequency (n)	Percentage (n/N)
United States of America	10	40.00%
Congo	4	16.00%
Nigeria	3	12.00%
United Kingdom	3	12.00%
Saudi Arabia	1	4.00%
Singapore	1	4.00%
Sudan	1	4.00%
Sweden	1	4.00%
United Arab Emirates	1	4.00%
Total (N)	25	100%

Most of the publications were in 2023 (20%). A total of 16% and 12% of the publications were in 2022 and 2019, respectively (Table 3). The total number of patients included in this systematic review was 758. However, the data for age were not available for 114 patients, so the mean age of 644 patients was 26.77±12.17 years (Table 4). Of the total 691 mentioned cases, 451 were males and 240 females, accounting for 65.27% and 34.73%, respectively.

**Table 3:** Publications according to years. The "n" refers to the frequency or number of presentations. The "n/N" signifies the formula for calculating the percentage, where "N" is the total number.

Year of publication	Frequency (n)	Percentage (n/N)
2024	2	8.00%
2023	5	20.00%
2022	4	16.00%
2021	2	8.00%
2019	3	12.00%
2013	1	4.00%
2010	1	4.00%
2007	1	4.00%
2006	1	4.00%
2005	2	8.00%
2004	2	8.00%
2003	1	4.00%
Total (N)	25	100%

**Table 4:** Gender of the reported cases. The "n" refers to the frequency or number of presentations. The "n/N" signifies the formula for calculating the percentage, where "N" is the total number.

Gender	Frequency (n)	Percentage (n/N)
Male	451	65.27%
Female	240	34.73%
Total (N)	691	100%

Of the total 715 reported cases, 701 (98.04%) were found to have no preexisting or unspecified conditions. Three cases accounting for 0.42% were observed to have hepatitis C (Table 5). Animal source/human contact was the most frequent mode of transmission, accounting for 87.05% of the total cases. The mode of transmission was unknown in 9.52% of patients. A small percentage (3.28% of patients) had transmission via sexual intercourse (Table 6).

**Table 5:** Concomitant conditions in reported cases of monkeypox. The "n" refers to the frequency or number of presentations. The "n/N" signifies the formula for calculating the percentage, where "N" is the total number. GERD: gastroesophageal reflux disease

Concomitant conditions	Frequency (n)	Percentage (n/N)
None/unspecified	701	98.04%
Hepatitis C	3	0.42%
High-risk sexual behaviour	1	0.14%
Primary syphilis infection	1	0.14%
Chronic back pain	1	0.14%
HIV infection	1	0.14%
Varicella zoster virus	1	0.14%
Pregnancy	1	0.14%
Hydrocephalus	1	0.14%
Lupus nephritis	1	0.14%
Hemophilia	1	0.14%
Recipient of bone marrow transplant	1	0.14%
GERD	1	0.14%
Total (N)	715	100%

**Table 6:** Modes of transmission of monkeypox. The "n" refers to the frequency or number of presentations. The "n/N" signifies the formula for calculating the percentage, where "N" is the total number.

Mode of transmission	Frequency (n)	Percentage (n/N)
Animal source/human contact	558	87.05%
Unknown	61	9.52%
Sexual intercourse (vaginal/anal)	21	3.28%
Infected hospital equipment	1	0.16%
Total (N)	641	100%

Fatigue/malaise (34.37%) was the most common presentation. Fever and headache were reported in 31.27% and 29.84%, respectively (Table 7). The total number of presenting complaints was 774, which is higher than the total number of patients (758), as some patients had more than one presenting complaint. The mean duration of neurological symptoms of 52 documented patients is 20.83 days.

**Table 7:** Presenting neurological complaints in reported cases. The "n" refers to the frequency or number of presentations. The "n/N" signifies the formula for calculating the percentage, where "N" is the total number.

Presenting complaint (neurological)	Frequency (n)	Percentage (n/N)
Fatigue/malaise	266	34.37%
Fever	242	31.27%
Headache	231	29.84%
Myalgia	8	1.03%
Back pain	4	0.52%
Disorientation/confusion	4	0.52%
Urinary retention	3	0.39%
Dysphagia	3	0.39%
Seizure	3	0.39%
Altered mental status	2	0.26%
Ear pain	2	0.26%
Vomiting/nausea	2	0.26%
Complete paralysis	1	0.13%
Facial drop	1	0.13%
Priapism	1	0.13%
Agitation	1	0.13%
Total (N)	774	100%

Limb weakness/numbness was the most common finding (20.83%). Urinary retention was found in 8.33%, and meningeal irritation was found in 6.25% of the patients. Bladder/bowel incontinence and areflexia were seen in 6.25% of cases, each (Table 8).

**Table 8:** Examination findings of reported cases. The "n" refers to the frequency or number of presentations. The "n/N" signifies the formula for calculating the percentage, where "N" is the total number.

Examination findings	Frequency (n)	Percentage (n/N)
Limb weakness/numbness	10	20.83%
Urinary retention	4	8.33%
Signs of meningeal irritation	3	6.25%
Bowel/bladder incontinence	3	6.25%
Areflexia/depressed reflexes	3	6.25%
Slurred speech/dysarthria	2	4.17%
Somnolence	2	4.17%
Facial weakness	2	4.17%
Altered sensations	2	4.17%
Miosis	1	2.08%
Hoffmann sign	1	2.08%
Neural plantar reflex	1	2.08%
Psychomotor deceleration	1	2.08%
Guillain-Barre syndrome	1	2.08%
Mastication weakness	1	2.08%
Bulbar weakness	1	2.08%
Neck muscle weakness	1	2.08%
Neuropathic pain	1	2.08%
Photosensitivity	1	2.08%
Hyperthermia	1	2.08%
Irritability	1	2.08%
Pupillary dilation	1	2.08%
Rigidity	1	2.08%
Optic disc edema	1	2.08%
Diminished corneal, gag reflex	1	2.08%
Ankle clonus	1	2.08%
Total (N)	48	100%

Vesiculopapular rash developed in 48.57% of the cases. About 15.91% had developed a sore throat and symptoms of pharyngitis. Lymphadenopathy was present in 14.10% of the cases (Table 9). Out of a total of 167 cases, 70.06% were coinfected with chickenpox, 23.95% with varicella zoster virus, and 3.59% with syphilis (Table 10).

**Table 9:** Associated non-neurological findings in reported cases. The "n" refers to the frequency or number of presentations. The "n/N" signifies the formula for calculating the percentage, where "N" is the total number.

Associated non-neurological findings	Frequency (n)	Percentage (n/N)
Vesiculopapular rash (genitals, lips, face, extremities )	696	48.57%
Pharyngitis/sore throat	228	15.91%
Lymphadenopathy	202	14.10%
Anorexia	109	7.61%
Cough	107	7.47%
Pruritus	58	4.05%
Genital lesions	8	0.56%
Flu-like symptoms/coryza	4	0.28%
Tonsillar hypertrophy	3	0.21%
Dyspnea/airway obstruction	3	0.21%
Oral ulcers	2	0.14%
Diarrhea	2	0.14%
Nausea/vomiting	2	0.14%
Nodular skin lesions	2	0.14%
Abdominal pain	1	0.07%
Proctitis	1	0.07%
Bleeding orifices	1	0.07%
Hepatosplenomegaly	1	0.07%
Blepharitis	1	0.07%
Eschar	1	0.07%
Night sweats	1	0.07%
Total (N)	1433	100%

**Table 10:** Coinfections with other pathogens. The "n" refers to the frequency or number of presentations. The "n/N" signifies the formula for calculating the percentage, where "N" is the total number. VZV: varicella zoster virus

Coinfections	Frequency (n)	Percentage (n/N)
Chickenpox	117	70.06%
VZV	40	23.95%
Syphilis	6	3.59%
HIV	2	1.20%
Herpes	1	0.60%
Malaria	1	0.60%
Total (N)	167	100%

The most common finding in CSF was normal glucose level corresponding to 26.32%. Raised white blood cells were seen in 21.05% of the cases. Normal protein levels were seen in 13.16% of the cases. Raised protein levels were present in 10.53% of the cases (Table 11).

**Table 11:** Cerebrospinal fluid examination findings. The "n" refers to the frequency or number of presentations. The "n/N" signifies the formula for calculating the percentage, where "N" is the total number. PCR: polymerase chain reaction; ELISA: enzyme-linked immunosorbent assay

CSF examination	Frequency (n)	Percentage (n/N)
Normal glucose	10	26.32%
Raised WBCs	8	21.05%
Normal protein	5	13.16%
Raised protein	4	10.53%
PCR positive	2	5.26%
Mononuclear cells	2	5.26%
Oligoclonal bands	2	5.26%
Normal WBCs	1	2.63%
Lymphocytes	1	2.63%
Pleocytosis	1	2.63%
Polymorphonuclear cells	1	2.63%
IgM ELISA positive	1	2.63%
Total (N)	38	100%

T2 hyperintensities and fluid-attenuated inversion recovery (FLAIR) hyperintensities were the most common finding comprising 18.75%. Diffuse edema, restricted diffusion, enhancement, and non-enhancing lesions were noticed in 9.38% of the cases, each (Table 12).

**Table 12:** Magnetic resonance imaging findings in the reported cases. The "n" refers to the frequency or number of presentations. The "n/N" signifies the formula for calculating the percentage, where "N" is the total number. FLAIR: fluid-attenuated inversion recovery; DWI: diffusion-weighted imaging

MRI findings	Frequency (n)	Percentage (n/N)
T2 hyperintensities	6	18.75%
FLAIR hyperintensities	6	18.75%
Diffuse edema	3	9.38%
Restricted diffusion	3	9.38%
Enhancement	3	9.38%
Non-enhancing lesions	3	9.38%
DWI hyperintensity	2	6.25%
Inflammation	2	6.25%
Patchy low apparent diffusion coefficient signal	2	6.25%
Normal findings	1	3.13%
Non-specific lesions	1	3.13%
Total (N)	32	100%

The brainstem (pons or medulla) was involved in 18.60% of the cases. Cerebral cortices, white matter and thalamus were involved in 16.28% and 13.95% of the cases, respectively. Post-treatment outcome is not mentioned for 692 patients (Table 13).

**Table 13:** Parts of the brain parenchyma involved. The "n" refers to the frequency or number of presentations. The "n/N" signifies the formula for calculating the percentage, where "N" is the total number.

Site of brain involved	Frequency (n)	Percentage (n/N)
Brainstem (pons, medulla)	8	18.60%
Cerebral cortices, white matter	7	16.28%
Thalamus	6	13.95%
Basal ganglia	4	9.30%
Splenium of corpus callosum	3	6.98%
Cerebellum	3	6.98%
Frontal cortex	3	6.98%
Subcortical region	2	4.65%
Cerebellar peduncle	2	4.65%
Internal capsule	2	4.65%
Cingulate gyrus	1	2.33%
Insula	1	2.33%
Parietal lobe	1	2.33%
Total (N)	43	100%

Among the 630 reported patients, 94.13% of the cases survived the course of infection. About 3.17% of the patients died during the course of infection. The outcome is not known for 2.70% of patients. Fifteen patients' follow-up is mentioned with a mean of 1.36 months (Table 14).

**Table 14:** Outcome of monkeypox infection. The "n" refers to the frequency or number of presentations. The "n/N" signifies the formula for calculating the percentage, where "N" is the total number.

Outcome	Frequency (n)	Percentage (n/N)
Alive	593	94.13%
Dead	20	3.17%
Unknown	17	2.70%
Total (N)	630	100%

Of the total 11 patients, 27.7% had treponemal antibodies and 18.18% were found with oligoclonal bands (Table 15). Medical treatment was documented for 45 patients. The treatment protocol was not mentioned for 713 patients. Sixty-six patients improved with the course of treatment. Antibiotics were given to 34.61% of patients for management of monkeypox. Tecovirimat and steroids were given to 10.26% of patients, each. Intravenous immunoglobulins were given in 8.97% of the cases (Table 16).

**Table 15:** Antibodies positivity. The "n" refers to the frequency or number of presentations. The "n/N" signifies the formula for calculating the percentage, where "N" is the total number. ADEM: acute disseminated encephalomyelitis; TPPA: Treponema Pallidum Particle Agglutination; ASMA: anti-smooth muscle antibodies; HHV: human herpesvirus; TBE: tick-borne encephalitis

Antibodies positivity	Frequency (n)	Percentage (n/N)
Treponemal antibodies	3	27.27%
Oligoclonal bands	2	18.18%
TPPA antibodies	1	9.09%
IgG TBE	1	9.09%
IgG *Borrelia burgdorferi*	1	9.09%
ASMA antibodies	1	9.09%
IgM HHV-2	1	9.09%
ADEM	1	9.09%
Total (N)	11	100%

**Table 16:** Course of medical treatment. The "n" refers to the frequency or number of presentations. The "n/N" signifies the formula for calculating the percentage, where "N" is the total number. IVIG: intravenous immunoglobulin

Medications	Frequency (n)	Percentage (n/N)
Antibiotics	27	34.61%
Tecovirimat	8	10.26%
Steroids (prednisolone, methylprednisolone, dexamethasone)	8	10.26%
IVIG	7	8.97%
Acyclovir	6	7.69%
Plasmapheresis	5	6.41%
Brincidofovir	4	5.13%
Valacyclovir	3	3.85%
Cidofovir	2	2.56%
Rituximab	2	2.56%
Antiepileptic	2	2.56%
Emtricitabine	1	1.28%
Tenofovir	1	1.28%
Opiate	1	1.28%
Neuropathic drugs	1	1.28%
Total (N)	78	100%

Genetic Variations

There was about 42 mutation difference from a sample taken during the 1971 human monkeypox outbreak in Nigeria [20]. The entire hemagglutinin gene sequence of the novel North American isolates was identical to hemagglutinin genes obtained from monkeypox virus isolated from patients in West Africa and from non-human primates in primate colonies [22].

Genetic analysis encompassing an 81,137 nucleotide section of the genome, which spans a region between the E9L and A24R loci, indicated that the 2010 isolate is highly similar to the virus found in the hospital-associated outbreak of monkeypox that occurred in Impfondo in 2003 [12]. Within the coding region examined (E9L-A24R), there is only one nucleotide difference between the two isolates that is predicted to result in an amino acid change [2].

The hemagglutinin gene (942 bp) of Sudan viruses was identical to that of the MPXV Congo Basin strain MPXV2003_DRC and MPXV1979_Zaire and had changes compared with that of MPXV West Africa strains MPXV2003_US and MPXV_WalterReed267 [18].

Discussion

Since January 2023, according to the Centers for Disease Control and Prevention (CDC), the Democratic Republic of the Congo (DRC) has reported more than 27,000 suspected MPX cases and more than 1,300 deaths leading to a sense of alarm [31]. On August 14, 2024, the World Health Organization determined that the upsurge of MPX in countries of Africa constitutes a Public Health Emergency of International Concern (PHEIC) under the International Health Regulations (2005) [32]. Although the spread is mostly limited to African countries, it is important that the disease should be contained and preventive measures should be undertaken to reduce the likelihood of a worldwide pandemic as we have witnessed in the case of COVID-19.

The mechanism of neurological involvement is variable among different pathogens with variable presentation of patients leading to diagnostic difficulty. According to a recent systematic review by Deb et al., headache, stroke, Guillain-Barré syndrome (GBS), seizures, and nerve palsies were the most common manifestations of COVID-19, while in MPX, headache and encephalitis were mostly seen [33]. Khan et al. conducted a recently published systematic review in which they reported headache (48.84%), myalgia (27.50%), fatigue (17.73%), and photophobia (4.43%) as the most common presentations in a certain proportion of MPX patients [34]. Our review had a slightly different spectrum of clinical presentations, with 34.37% reporting symptoms of fatigue/malaise, followed by fever and headache accounting for 31.27% and 29.84%, respectively. Since the recently reported cases are included, this is an update of the review by Khan et al. which could imply that patients presenting with neurological manifestations of MPX essentially have a similar prodrome of initial neurological symptoms with slight variations [34]. Furthermore, the severity of viral encephalitis depends on the response of microglia, and innate and adaptive immunity [35]. It is proposed that prolonged chronic microglial activation as in viral encephalitis can eventually lead to increased tissue damage and neurological long-term sequelae [36]. The mean duration of neurological symptoms was about 20.83 days. This could explain why only a few patients presented with alarming symptoms and signs, such as paralysis, seizures, altered mental status, disorientation, dysphagia, bowel/bladder incontinence, etc.

McEntire et al. in their scoping review of different WHO epidemics and pandemics concluded that in four pathogens namely monkeypox, Middle East respiratory syndrome, smallpox, and SARS-CoV-2, the question of direct neurotropism remains elusive. On the other hand, chikungunya, influenza, Lassa fever, COVID-19, smallpox, tularemia, and Zika virus have shown evidence of parainfectious neurological syndromes in addition to direct neurotropism [37]. Moreover, it is difficult to isolate the causative agent when there is a significant overlap among different viral infection manifestations. In our review, out of a total of 167 cases, 70.06% had chickenpox, 23.95% had Varicella zoster virus, and 3.59% were found with syphilis. Although limited patients had coexisting infections, this adds to the discrepancy in diagnosis as neurological symptoms can overlap. On the contrary, according to the NHS, the rash of MPX is similar to chickenpox. Therefore, patients presenting with a provisional diagnosis of chickenpox could be cases of MPX [38]. Hence, the true neurotropism of MPX is still under debate.

Currently, the exact transmission routes of the MPX virus are not known. Previous studies on animal subjects have suggested the two most likely routes, namely the olfactory epithelium route and hematogenous spread by monocyte/macrophage transmission [2]. This would explain why there is a significant onset in neurological manifestations as olfactory epithelium is the direct route to the brain. The definitive reservoir has not been well established. Spread between humans is possible but occurs at lower rates [37]. The reported mode of transmission of MPX is consistent with compiled data with as high as 87.05% of patients contracting from infected animals or other humans. Sexual intercourse is another significant emerging source of transmission which means in addition to direct skin contact, the exchange of body fluids can also cause the spread of the virus.

MPX is diagnosed based on epidemiological, clinical, and laboratory findings [1]. Although specific clinical features are sufficient to suspect or even confirm a case of MPX, ambiguity occurs when the virus infiltrates the brain. MPX-associated encephalitis can infect the central nervous system retrogradely via nerve terminals or hematogenous dissemination [5]. Nevertheless, there is a diagnostic dilemma, especially in cases that present only with neurological signs. Cerebrospinal fluid has predominantly polymorphonuclear pleocytosis with normal glucose and protein levels. There may be lymphocytosis which may decrease and primarily include lymphocytes [2,4]. Our review illustrated slightly different findings, with 21.05% of CSF examinations showing raised white blood cells, raised protein levels in 10.53%, and normal glucose levels in 26.32%. However, these findings are not definitive since they represent only a small number of patients. The magnetic resonance imaging data shows diffuse edema, meningeal amplification, and signal abnormalities in the thalamus and parietal cortex [4]. Additionally, hyperintensity in the thalamus, brainstem, and right posterior parietal cortex consistent with mixed cytotoxic and vasogenic brain edema is also seen in MPX encephalitis [2]. In coherence with reported findings, 18.75% of the cases were reported with T2 hyperintensities and fluid-attenuated inversion recovery (FLAIR) hyperintensities separately in our review. Similarly, most cases (18.60%) had brainstem involvement. A total of 16.28% had cerebral cortices and white matter involvement, while 13.95% had thalamus involvement. With limited evidence, it is premature to say that MPX involving the CNS can be differentiated effectively from other viral encephalitis on neuroimaging [37].

The management protocol has mostly been individualized according to symptoms and disease severity. The majority of cases require conservative, symptomatic management, and antiviral therapy, with some serious cases complicating into encephalitis requiring intubation and ventilatory support. Usual treatment protocols include cidofovir, tecovirimat, corticosteroids, immunoglobulins, and vaccines [3]. Some next-generation vaccines, including Modified Vaccinia Ankara-Bavarian Nordic, ACAM2000, and LC16m8 vaccine, were approved with limited use in high-risk individuals, but they are not feasible in every setting [34]. ACAM2000 has the potential to cause a range of other serious adverse events, thus limiting its use [1]. With the data isolated in the present review, many patients were inadvertently given antibiotics for the management of MPX, which is probably to prevent superimposed infections, especially in immunocompromised individuals. A total of 10.26% were given tecovirimat and steroid therapy, respectively. Intravenous immunoglobulins were given in 8.97% of cases. It is difficult to interpret the long-term effectiveness of this treatment protocol. Nevertheless, overall survival was 94.13%, but follow-up of only 15 patients was mentioned with a mean follow-up of 1.36 months. The question of MPX causing long-term neurological complications is still an enigma.

**Figure 2:**
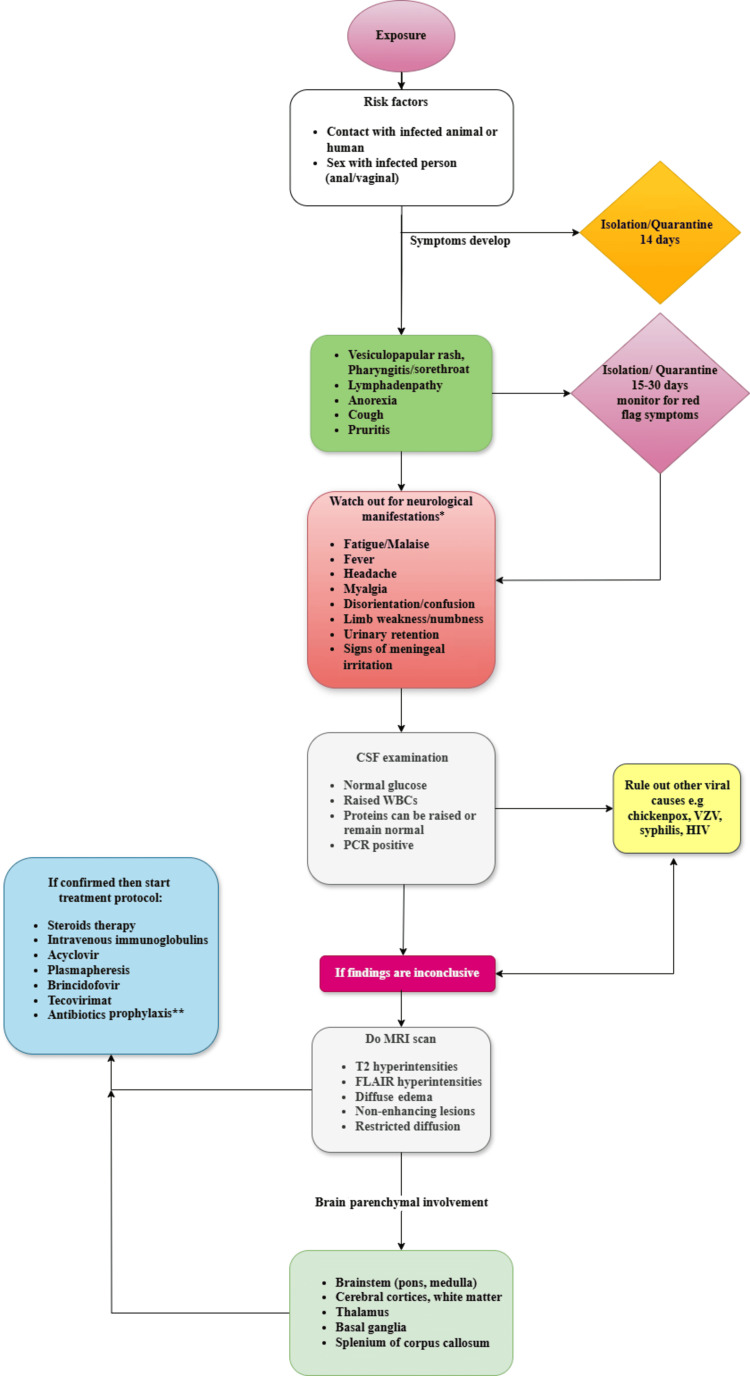
Managerial algorithm for monkeypox infection with neurological manifestations (author-proposed version). *Most probable neurological manifestation according to reported cases. **Antibiotic prophylaxis should be started on concomitant bacterial infection with monkeypox Infection in suspected cases. The image is created by the author/contributor Arham Amir and Hafiz Sohail of this study. PCR: polymerase chain reaction; FLAIR: fluid-attenuated inversion recovery

Much information is available on the epidemiology, presentations, diagnosis, treatment options, and clinical outcomes of MPX, but we have tried our best to delineate the neurological manifestations of the MPX virus and its management (Figure 2).

Limitations

There were certain limitations of this systematic review. Firstly, this review focused on signs and symptoms involving the brain only. Secondly, we only sequestered the data from articles available on PubMed Central and Google Scholar. Thirdly, we encountered gaps in data acquisition, particularly regarding patient treatment information, which was frequently absent and contributed to the review's overall incompleteness. Additionally, there was insufficient data on patient follow-up, further limiting the scope of our findings.

## Conclusions

This systematic review represents a tiny fragment of what we know about MPX and its neurological sequelae. Even though a long road exists in understanding brain pathophysiology itself, it is still a step forward for the better identification of patients suffering from neurological symptoms in MPX. The neurotropism of MPXV is still debated and needs further exploration. Neurologists and neurosurgeons can implement new treatment protocols and can generate new guidelines for MPX management. Early identification and interventions by healthcare providers can significantly impact the trajectory of MPX spread. A special focus on the long-term effects of MPXV on the brain can help reveal the exact mechanisms of neuroinvasion.

The COVID-19 pandemic offers valuable lessons for future preparedness and response. Therefore, there is a need for strong public health systems, and global cooperation to address future crises. The interconnection of physicians and clinicians throughout the world can help to prevent such spread in the distant future.
